# The relationship between depression and sexual health service utilization among men who have sex with men (MSM) in Côte d'Ivoire, West Africa

**DOI:** 10.1186/s12914-019-0186-6

**Published:** 2019-03-05

**Authors:** Mark B. Ulanja, Carrie Lyons, Sosthenes Ketende, Shauna Stahlman, Daouda Diouf, Abo Kouamé, Rebecca Ezouatchi, Amara Bamba, Fatou Drame, Ben Liestman, Stefan Baral

**Affiliations:** 1 Center for Public Health and Human Rights, Department of Epidemiology, Johns Hopkins Bloomberg School of Public Health, Baltimore, E7146, 615 N. Wolfe Street, Baltimore, MD 21205 USA; 2 Enda Santé, Dakar, Senegal; 3 Enda Santé, Abidjan, Côte d’Ivoire; 4 Programme National de Lutte contre le Sida, Ministère de la Santé et de la Lutte contre le Sida, Abidjan, Côte d’Ivoire

**Keywords:** Depression, Sexual health services, Men who have sex with men, Epidemiology, Gender, HIV, Cote D’Ivoire

## Abstract

**Background:**

In Cote D’Ivoire, there has been limited coverage of evidence-based sexual health services specifically supporting men who have sex with men (MSM). To date, there has been limited study of the determinants of engagement in these services including multiple intersecting stigmas and depression.

**Methods:**

1301 MSM aged 18 years and older, were recruited using respondent-driven sampling in Abidjan, Yamoussoukro, Gagnoa and Bouake, Cote d’Ivoire from January 2015 to October 2015. Inclusion criteria included anal sex with another man in the past 12 months were to complete a structured questionnaire including the Patient Health Questionnaire (PHQ)-9 to screen for depression. Chi-Square tests were used to test difference in healthcare utilization across variables, and multiple logistic regression was used to test the association between depression and health care utilization represented by HIV and sexually transmittable infection testing and treatment.

**Results:**

Depression (aOR:1.40, 95% CI: 1.07–1.84), being aged 25–29 years (aOR:1.84, 95% CI: 1.11–3.03),unemployed (aOR:0.64, 95% CI: 0.42–0.98), being a student (aOR:0.67, 95% CI: 0.48–0.96), being identified as male (aOR:0.44, 95% CI: 0.29–0.67), and identifying as homosexual (aOR:0.74, 95% CI:0.56–0.99) were significantly associated with utilization of sexual health care services in the final multivariable model. Healthcare enacted stigma (aOR: 1.55, 95% CI: 1.03–2.33) was associated with utilizing sexual health care services, but perceived healthcare stigma, social stigma and family stigma were not.

**Conclusion:**

Given higher levels of depressive symptomatology among those engaging in sexual health care services, this engagement represents an opportunity for service integration which may have synergistic benefits for both sexual and mental health. Moreover, MSM in Cote D’Ivoire who had engaged in sexual health services were more likely to report having experienced health-care enacted stigma. Taken together, these results reinforce the need for stigma mitigation interventions to support sustained engagement in HIV prevention, treatment and care services as a means of reducing health disparities among MSM in Cote d’Ivoire.

## Background

A leading cause of disability and global burden of disease is depression affecting more than 350 million worldwide [1], yet in many Sub-Saharan African settings fewer than one in ten individuals have access to adequate mental care and treatment [2, 3]. However, the prevalence of depression is not evenly distributed, with the prevalence of depression among MSM estimated to range from 44 to 58% in Southern Africa or about 4 times the prevalence in broader population [4–6]. While literature is rich with research on depression among MSM in high income countries, the same cannot be said of low and middle income countries [7] such as Cote d’Ivoire, or more broadly across West Africa. Studies in Tanzania and South Africa have estimated the prevalence of depression among MSM to be 46.3 and 44% respectively [5, 7]. The prevalence of depression in Cote d’Ivoire is estimated to be 20% among adults receiving psychiatric services [8]. However, there are no reliable data on depression in the broader population. Similarly, neighboring West African countries have limited data on depression, with only some data for specific demographic groups including university students (39.2%) [9] in Ghana and Cote d’Ivoire [9, 10].

Previous studies have consistently shown that MSM may be at higher risk for depression secondary to minority stress conceptualized as the social environmental stressors associated with being a sexual minority [11, 12]. Minority stress has consistently been shown to be induced by hostile and homonegative environments that precipitate multiple forms of stigma and discrimination including harassment, suboptimal health care services, violence, and even murder [11–13]. Health disparities potentiated by minority stress are more likely to be pronounced in areas where same-sex practices among MSM are criminalized [11] including many countries across Sub-Saharan Africa.

Depression can result in internalized stigma and limited self-efficacy, which may lead to impaired physical, social, and emotional health [14–16]. In particular, MSM who are depressed may be more likely to use alcohol or to have an alcohol use disorder [17]. Moreover, alcohol use associated with depression has been shown to increase sexual risk behaviors and human immunodeficiency virus(HIV) infection among young adults in Sub-Saharan Africa [18]. Depression impairs quality of life, potentially adversely impacting the need to use health care services [19, 20]. Depression and substance use have also been found to be an outcome of sexual violence affecting MSM in low and middle income countries [21–24]. In a study conducted among MSM in Mexico, it was found that 39% reported sexual violence, and the authors emphasized the importance of preventing sexual violence and stigma to mitigate the development of adverse mental health and physical health outcomes [25].

Across Sub-Saharan Africa, consistent data have demonstrated concentrated HIV epidemics among key populations including MSM, even in the context of more generalized epidemics [16, 26, 27]. In West and Central Africa, HIV and other sexually transmitted infection (STI) prevalence among MSM range from 5 to 10 times higher than all reproductive aged adults, and HIV prevalence as high as 50% among MSM in some countries [20, 28, 29]. Potentiating risks of HIV among MSM are the challenges that many MSM face in accessing health care services including stigma and discrimination and overt violence as a result of their sexual practices [16, 20, 30–32]. Stigma and discrimination are also strongly related to depression, which can negatively impact health outcomes [14, 31]. In the Gambia, MSM experiencing stigma in health care settings had three times the odds of significantly avoiding and delaying health care utilization, as well as associated poorer health outcomes [33]. In Cote d’Ivoire specifically, HIV prevalence is estimated at 3.7% [21] among all reproductive age adults, but estimated to be 18.0% among MSM [34] and up to 50.0% among MSM engaging in commercial sex work [28].

Same sex practices are not criminalized in Cote d’Ivoire, and the Ministry of Health defined MSM as a priority population in the National Strategic Plan for the Fight against HIV/AIDS 2011–2015 [34]. For evidence-based strategies to work and ultimately reduce the incidence of HIV and STIs, and improve sexual health wellbeing, factors which may affect the utilization of sexual health care services among MSM should be explored. Thus, the objective of these analyses is to assess the relationship between depression and stigma relating to the utilization of sexual health care services among MSM in Cote d’Ivoire.

## Methods

### Participants

Data were obtained from a cross-sectional study (*N* = 1301) conducted in 5 cities in Cote d’Ivoire, including Abidjan (*n* = 351), Bouake (n = 351), Gagnoa (*n* = 150), Agboville (*n* = 200) and Yamoussoukro (*n* = 250). Individuals were eligible to participate in the study if they were aged ≥18 years and reported having anal intercourse (either receptive or insertive) in the past 12 months. Participants were required to have lived in the country for the past 3 months, and be able to give informed consent prior to participating in the study. MSM were excluded if they demonstrated mental incapacity, were under the influence of substances at the time of participation, or had any other illness preventing comprehension of the study procedures and informed consent.

Participants were recruited using respondent-driven sampling (RDS) [35, 36], which is a peer-driven chain referral sampling method useful for reaching hidden populations such as MSM. Across all of the sites, 9 MSM initial recruits (seeds) were purposively chosen by the investigators as people who were sociometrically well-connected and interested in participating in the study. Seeds completed a behavioral survey, biological testing, and training to become recruiters. They were then given three coupons to recruit other MSM. Participants completed a study visit with a trained MSM or MSM-friendly interviewer, who administered a structured survey instrument including modules on demographics, depression [37, 38], alcohol use [39, 40], STIs, and access to health care services. Participants also completed biological testing. Out of the nine initial seeds at all sites, nine recruited participants in Abidjan, eight in Bouake, five in Gagnoa, six in Agboville and seven in Yamoussoukro. Participants were reimbursed the equivalent of approximately 4 USD for their time and travel to the study site. In addition, recruiters were compensated the equivalent of 2 USD for each eligible participant they recruited into the study. Gender identity of the study participant was collected using the two-step gender assessment [41].

### Stigma

Several types of sexual behavior-related stigma were measured. MSM’s experience of each broad area of stigma (health care, family/friend and social stigma) was assessed by using responses “yes” or “no” to each individual item. Each area was assessed for association with the outcome variable by using the sum of the total number of “yes” and “no” responses [4, 30]. Family- or friend-related stigma included feeling excluded from family activity, hearing discriminatory remarks from family, or feeling rejected by friends because of having sex with men. Health care stigma included being afraid to use health services, avoiding health services, feeling mistreated in a health center because of having sex with men, or hearing a healthcare worker make discriminatory remarks or gossip about MSM [37, 42]. Social stigma included feeling like police refused to protect, being arrested on charges of homosexuality, ever being jailed, being scared to walk in public, being verbally harassed, blackmailed, or physically hurt because of having sex with men. The cumulative, dichotomized “yes”/“no” responses, for each category of stigma [43] (i.e., family/friend, health care and social stigma) were used in the logistic regression models. Health care stigma was further categorized using as perceived or enacted.

Finally, forced sex was measured by asking the participant if they have ever been coerced into having anal, vaginal or oral penetration against their will. Responses were dichotomized into “yes”/“no”.

### Depression and alcohol use

Depression and depressive symptoms were measured using the Patient Health Questionnaire (PHQ-9) [38], which has been shown to have high sensitivity and specificity among adults in primary care services in Sub-Saharan Africa [44, 45]. It was dichotomized into depressed (> 4) and not depressed (0–4) [46]. A cutoff of 5 for minimal depression was chosen [7, 37, 38, 44] to increase sensitivity and to capture respondents who may have any level of depression irrespective of severity [7]. This cutoff point has been shown in validation studies to have a sensitivity of 83% and a negative predictive value of 98% in Sub-Saharan African adults for detecting major depressive disorder [45].

Alcohol use was measured using the Alcohol Use Disorders Identification Test (AUDIT), which has been shown to be valid across low and middle-income countries [39, 40, 47]. The tool has been shown to have sensitivity (99.3%) and specificity (77.8%) [39, 48] among populations in African countries.

### Utilization of sexual health care services

The primary outcome was utilization of sexual health care services, measured with two indicator variables: 1) tested for sexually transmitted infections or received treatment for sexually transmitted diseases/infection (STI) in the past 12 months (yes/no), and 2) tested for HIV in the past 12 months (yes/no). These indicators of health care utilization were chosen because of the important role they play in reducing HIV infection [34, 49]. The former included MSM who experienced symptoms of STIs and sought care from health care workers, and were subsequently treated. MSM who reported symptoms but either self-treated, went to a traditional healer, or did nothing about their symptoms were grouped as “not treated”. HIV testing was measured by asking participants if they have ever completed HIV test at a health care facility in the past 12 months. The responses were recorded as “yes”/“no”, depending on when the test was done (≤12 months as “yes”, and “no” for otherwise). Participants who indicated “yes” to either indicator were grouped as having a positive result for the final outcome measure for utilization of sexual health care services.

### Statistical analyses

Data from the 5 study sites were combined and analyzed using Stata 14.0 (Stata Corp, College Station, Texas). Chi-square tests were used to compare difference in health care use among various socio-economic indicators and other variables. Logistic regression models were used to examine the association between the outcome measure (utilization of sexual health care services) and covariates such as depression, alcohol intake, employment, educational status and stigma, in both bivariate and multivariable models. For the HIV test variable, participants who had an HIV test more than one year prior (2.4%, 15/633) were not included in the final model, because one of the inclusion criteria was having anal sex in past 12 months.

There were two final models: Model 1 included depression, age, education, employment status, residence (living alone or with spouse or other MSM), gender, sexual orientation, forceful engagement in sex, and condom use. Model 2 included variables in model 1 and stigma variables. Two separate models were used because stigma has been shown to be on the causal pathway to depression and thus depression may be acting as a mediator between stigma and utilization of sexual health care services [50]. These variables were included because they may be associated sexual behavior among MSM, and health outcomes in general [15, 20, 34, 50–53]. The impact of alcohol consumption was not significant in both bivariate analysis and adjusted models, hence was excluded from the final models. Variance inflation factor (VIF) was used to check for collinearity and determined that collinearity was not of significant concern (VIF < 10) [54, 55].

RDS sampling probabilities were adjusted for in the final models [56]. The final models selected were checked for goodness of fit using Hosmer- Lemeshow Goodness-of-Fit test and both models were determined to have appropriate fit (Model 1 Hosmer-Lemeshow chi2(8) = 6.06, *p* = 0.64 and Model 2 (Hosmer-Lemeshow chi2(8) =6.19, *p* = 0.63)) [57]. Finally missing data for the variables of interest was assessed and complete case analysis was used to handle missing data. Any missing data was not missing at random (MAR), and hence there was no need to use imputation technique in analyzing these data.

## Results

### Characteristics of study sample

The age of participants ranged from 18 to 54 years. The median age was 23 years with an interquartile range (IQR) of 21–26 years (Table 1). The largest proportion of participants were between 20 and 24 years old (49.1%, 639/1301), and the fewest were aged ≥30 years (10.3%, 134/1301). Most were students (50.9%, 662/1300), employed 35.7%(464/1300) and 13.4%(174/1300) unemployed.

**Table 1:** Prevalence of variables of interest among MSM in Cote d’Ivoire, *N* = 1301

Variable	*N* (%)	RDS adjusted (%)
Depression		
Depressed	624 (48.0)	41.9
Not depressed	677 (52.0)	58.1
Depression categorization		
Not depressed (0–4)	677 (52.0)	58.1
Mild (5–9)	377 (29.0)	25.3
Moderate (10–14)	188 (14.5)	12.3
Moderately severe (15–19)	45 (3.5)	3.5
Severe (20–27)	14 (1.1)	0.9
Age (years)		
< 20	154 (11.8)	14.8
20–24	639 (49.1)	50.4
25–29	374 (28.8)	27.2
≥ 30	134 (10.3)	7.6
Education		
Never	43 (3.3)	2.2
Primary or less	95 (7.3)	6.5
Secondary level	810 (62.3)	69.0
Above Secondary Level	353 (27.1)	22.4
Employment		
Yes	464 (35.7)	30.0
No	174 (13.4)	12.6
Student	662 (50.9)	57.5
Self-reported HIV test results		
Never tested	259 (19.9)	29.1
Tested negative in last 12 months	656 (50.4)	42.8
Tested negative in over 12 months	332 (25.5)	26.1
Positive	54 (4.2)	2.0
Alcohol use (score)		
Low risk (0–7)	418 (39.6)	37.0
Hazardous use (8–15)	443 (42.0)	45.3
Harmful use (16–19)	95 (9.0)	8.7
Almost Certainly Dependent use (≥20)	100 (9.5)	9.0
Place of residence		
Living with family/spouse	1040 (79.9)	79.8
Living alone	130 (10.0)	10.9
Living with friend/MSM/other	131 (10.1)	9.3
Gender		
Male	962 (73.9)	85.1
Transgender	338 (26.0)	14.9
Sexual orientation		
Bisexual	775 (59.6)	64.0
Homosexual	499 (38.4)	35.1
Heterosexual	27 (2.1)	0.9
Forced sex		
Yes	236 (18.1)	12.2
No	1065 (81.9)	87.8
Condom use in past 30 days		
No	193 (18.9)	17.0
Yes	827 (81.1)	83.0
Family/friends Stigma		
Felt Excluded from family activity	136 (10.5)	10.0
Discriminatory remark by family	433 (33.3)	28.7
Felt rejected by friends	299 (23.0)	23.6
Health care stigma		
Perceived		
Afraid to use health services	294 (22.6)	21.3
Avoided health service for STI/HIV	205 (15.8)	13.9
Enacted		
Felt not well treated	42 (3.2)	2.7
Heard healthcare worker gossiped	120 (9.2)	8.2
Social Stigma		
Felt police refused to protect	61 (4.7)	1.6
Arrested on charges of homosexuality	41 (3.2)	1.5
Ever jailed	20 (1.5)	0.9
Scared to walk in public	175 (13.5)	12.6
Verbally harassed	481 (37.0)	30.3
Blackmailed	245 (18.8)	11.5
Physically hurt	564 (43.4)	36.2

Self-reported HIV prevalence was 4.2% (54/1301), and 19.9% (259/1301) of participants had never tested for HIV. Abidjan had the highest percentage of participants who self-reported as HIV positive (61.1%, 33/54) and Gagnoa the least (1.9%, 1/54).

The prevalence of health care utilization in the last 12 months was 60.5% (787/1301). Of the participants who sought sexual health care services, 64.0% (830/1297) went to non-governmental facilities, while 13.8% (179/1297) went to either private or public facilities, but 22.2% (288/1297) had not sought sexual health care services prior. The prevalence of depression was 48.0% (624/1301), with 29.0% (377/1301) being mildly depressed, 14.5% (188/1301) moderately depressed, and 19.0% (247/1301) severely depressed.

Most participants identified their gender to be male 73.9% (962/1301), 22.2% (289/1301) identifying as a woman using the two-step gender assessment and 3.8% (50/1301) identifying as a transgender woman. In terms of sexual orientation, more than half identified as bisexual 59.6% (775/1301), 38.4% (499/1301) identifying as gay or homosexual and 2.1% (27/1301) as heterosexual.

Many of the participants were living with family or a spouse, 79.9% (1040/1301), and fewer were living alone (10.0%, 130/1301), or with friends or other MSM (10.1%, 131/1301). Participants experienced various forms of family-related stigma, such as feeling excluded from family activities (10.5%, 136/1301), hearing discriminatory remarks from family members (33.3%, 433/1301), and feeling rejected by friends (23.0%, 299/1301). Stigma in health care settings was also prevalent, with 22.6% reporting being afraid to seek health care services, 15.8% (205/1301) avoiding health care services, 3.2% (42/1301) feeling not treated well in a health care center, and 9.2% (120/1301) hearing a health care worker gossip about them. For social stigma, 4.7% (61/1301) felt like police refused to protect them, 13.5% (175/1301) felt scared to walk in public, 37.0% (481/1301) reported verbal harassment, 18.8% (245/1301) reported blackmail, and 43.4% (564/1301) reported being physically hurt because they have sex with men. The prevalence of being forced to engage in sexual activity was 18.1% (236/1301).

The percentage of MSM reporting alcohol consumption was 81.2% (1056/1301). More than one-third of drinkers (42.0%, 443/1056) were classified as risky/hazardous drinkers. Of the participants who engaged in anal sex in last 30 days, 18.9% (193/1020) did not use condoms, and 81.1% (827/1020) used condoms inconsistently.

### Bivariate associations with health care utilization

Utilization of sexual health care services in the past 12 months was significantly associated with depression (Odds Ratio [OR] = 1.69, 95% CI; 1.35–2.12); being aged 20–24 years (OR = 1.74, 95% CI; 1.22–2.48), aged 25–29 years (OR = 2.64, 95% CI; 1.80–3.88), and aged ≥30 years (OR = 2.44, 95% CI; 1.51–3.93) as compared with aged < 20 years; identifying as male vs transgender (OR = 0.59, 95% CI; 0.45–0.77), and identifying as heterosexual (OR = 2.81, 95% CI; 1.05–7.50) (Table 2). Furthermore, family related stigma (OR = 1.41, 95% CI; 1.12–1.77), health care enacted (OR = 1.69, 95% CI; 1.15–2.48)) and social stigma (OR = 1.38, 95% CI; 1.09–1.75) increased the odds of utilization of sexual healthcare services. Being unemployed (OR = 0.67, 95% CI; 0.47–0.96) and being a student (OR = 0.68, 95% CI; 0.53–0.87) were associated with reduced utilization of sexual health care services. Educational level was not significantly associated with utilization of sexual health care services.

**Table 2:** Bivariate Associations with Health Care Utilization

Characteristic	Health care use		RDS
	OR (95% CI)	*p*-value	OR (95% CI)
Depression			
Not depressed	Ref		
Depressed	1.69 (1.35–2.12)	<0.001	1.87 (1.32–2.66)
Age			
< 20	Ref		
20–24	1.73 (1.22–2.48)	**0.002**	2.63 (1.55–4.46)
25–29	2.64 (1.80–3.88)	**< 0.001**	3.73 (2.08–6.70)
≥ 30	2.44 (1.51–3.93)	**< 0.001**	3.45 (1.69–7.08)
Education			
Never	Ref		
Primary/less	0.79 (0.37–1.69)	0.546	0.50 (0.17–1.47)
Secondary	0.59 (0.31–1.14)	0.118	0.45 (0.19–1.09)
Above Secondary	1.21 (0.61–2.37)	0.591	0.89 (0.35–2.26)
Employment			
Yes	Ref		
No	0.67 (0.47–0.96)	0.027*~	0.72 (0.41-1.26)
Student	0.68 (0.53–0.87)	0.002*~	0.78 (0.53-1.15)
Alcohol use (score)			
Low risk (0–7)	Ref		
Hazardous use (8–15)	1.05 (0.80–1.38)	0.721	1.10 (0.72–1.69)
Harmful level (16–19)	1.10 (0.70–1.73)	0.694	1.09 (0.57–2.10)
Almost Certainly Dependent level (≥20)	0.97 (0.62–1.50)	0.877	0.93 (0.47–1.83)
Place of residence			
Alone	Ref		
Living with family/spouse	1.10 (0.76–1.59)	0.628	0.97 (0.56–1.69)
Living with friend/MSM/other	1.55 (0.94–2.58)	0.088	0.98 (0.45–2.12)
Gender			
Transgender	Ref		
Male	0.59 (0.45–0.77)	< 0.001*~	0.74 (0.48-1.15)
Sex orientation			
Bisexual	Ref		
Homosexual	0.90 (0.72–1.13)	0.371	0.70 (0.49–1.01)
Heterosexual	2.81 (1.05–7.50)	**0.039**	5.67 (1.57–20.48)
Forced sex			
No	Ref		
Yes	1.44 (1.07–1.94)	0.017*~	0.96 (0.56-1.63)
Condom use			
No	Ref		
Yes	1.19 (0.87–1.64)	0.275*	1.95 (1.18-3.21)
Family/friend			
No	Ref		
Yes	1.41 (1.12–1.77)	0.003*~	1.34 (0.94-1.91)
Health care- perceived			
No	Ref		
Yes	1.08 (0.83–1.39)	0.576	1.36 (0.94–1.97)
Health care – enacted			
No	Ref		
Yes	1.69 (1.15–2.48)	0.008*~	1.26 (0.72-2.20)
Social			
No	Ref		
Yes	1.38 (1.09–1.75)	0.007*~	1.38 (0.97-1.98)

*CI* confidence interval, *OR* odds ratio, *Family stigma* Felt Excluded from family activity, discriminatory remark by family, felt rejected, Perceived healthcare stigma: afraid to use health services, avoided health service for STI/HIV, Enacted healthcare stigma: Felt not well treated, heard healthcare worker gossiped, Social stigma: felt police refused to protect, arrested on charges of homosexuality, ever jailed, scared to walk in public, verbally abused, blackmailed, physically hurt

*~ not significant after Respondent Driven sampling (RDS) adjustment

*significant after RDS adjustment

Bolded *p*-values implies significance for RDS adjustment and for the model

### Adjusted associations with health care utilization

Depression (aOR:1.40, 95% CI: 1.07–1.84), being aged 25–29 years (aOR:1.84, 95% CI: 1.11–3.03), being unemployed (aOR:0.64, 95% CI: 0.42–0.98), being a student (aOR:0.67, 95% CI: 0.48–0.96), identifying as male (aOR:0.44, 95% CI: 0.29–0.67), and identifying as homosexual (aOR:0.74, 95% CI:0.56–0.99), were associated with utilization of sexual healthcare services (Table 3). Healthcare enacted stigma (aOR: 1.55, 95% CI: 1.03–2.33), was associated with utilization of sexual health care services, but perceived health care stigma, social stigma and family stigma were not. Health care enacted stigma, as well as social stigma showed associations with utilization of sexual health care services in bivariate models, but there was no independent association identified in the final model (Model 2) (Table 4). In a sensitivity analysis an additional model was used, in which depression was not included. This sensitivity analysis found that there was a relationship between increased utilization of sexual health services and health care enacted stigma (aOR = 1.67, 95% CI; 1.05–2.64). Condom use in past 30 days (aOR, 1.99, 95% CI: 1.21–3.30) was associated with utilization of sexual healthcare services after RDS adjustment.

**Table 3:** Final multivariable model 1: adjusted associations with USHCS

Variable	Adjusted Odds Ratio [95% Conf. Interval]	*P*-value	RDS [95% CI]
Depression	1.40 (1.07–1.84)	0.015	1.63(1.10–2.42)
Age			
20–24	1.41 (0.92–2.18)	0.119**	1.93 (1.04-3.60)
25–29	1.84 (1.11–3.03)	0.017	2.36 (1.15–4.84)
≥ 30	1.44 (0.77–2.69)	0.255	1.82 (0.75–4.42)
Education			
Primary/less	0.59 (0.22–1.63)	0.312	0.87(0.23–3.31)
Secondary	0.67 (0.27–1.65)	0.384	0.76 (0.25–2.35)
Above secondary	1.27 (0.50–3.24)	0.615	1.36 (0.40–4.59)
Employment			
No	0.64 (0.42–0.98)	0.041*	0.58 (0.29-1.17)
Student	0.67 (0.48–0.96)	0.027*	0.72 (0.43-1.20)
Residence			
Family/spouse	1.34 (0.86–2.08)	0.86 (2.08–0.196)	1.32(0.74–2.34)
Friend/msm/other	1.78 (0.96–3.28)	0.066	1.34 (0.58–3.08)
Gender			
Male	0.44 (0.29–0.67)	< 0.001	0.47 (0.23–0.98)
Sex orientation			
Homosexual	0.74 (0.56–0.99)	0.042	0.61 (0.39–0.94)
Heterosexual	2.00 (0.55–7.27)	0.291	3.98 (0.84–18.87)
Forced sex	0.94 (0.65–1.37)	0.754	0.64 (0.34–1.21)
Condom use (30 days)	1.35 (0.96–1.89)	0.081**	2.03 (1.23-3.37)

*Ref*: Depression: no depression, age: < 20, education: never, employment: yes, residence: alone, gender: transgender, sexual orientation: bisexual, forced sexual intercourse: no condom use: no

**= significant after RDS adjustment

*= not significant after RDS adjustment

**Table 4:** Final multivariable model 2: adjusted associations with utilization of sexual health care services (USHCS)

Variable	Adjusted Odds Ratio [95% Conf. Interval]	*P*-value	RDS [95% CI
Depression	1.36 (1.03–1.80)	**0.032**	1.59 (1.062.39)
Health care stigma			
Perceived	0.92 (0.68–1.25)	0.602	1.12 (0.71–1.75)
Enacted	1.55 (0.98–2.46)	0.064	1.13 (0.56–2.29)
Age			
20–24	1.44 (0.93–2.22)	0.102**	1.93 (1.04-3.60)
25–29	1.88 (1.14–3.10)	**0.014**	2.35 (1.14–4.83)
≥ 30	1.46 (0.78–2.74)	0.238	1.82 (0.75–4.40)
Education			
Primary/less	0.60 (0.22–1.64)	0.318	0.85 (0.23–3.13
Secondary	0.69 (0.28–1.68)	0.411	076 (0.25–2.35)
Above secondary	1.29 (0.50–3.31)	0.593	1.36 (0.41–4.56)
Employment			
No	0.63 (0.41–0.97)	0.037*	0.59 (0.29-1.18)
Student	0.67 (0.48–0.96)	0.027*	0.72 (0.43–1.20)
Residence			
Family/spouse	1.36 (0.87–2.11)	0.179	1.31 (0.74–2.33)
Friend/msm/other	1.77 (0.96–3.28)	0.069	1.35 (0.59–3.10)
Gender			
Male	0.44 (0.29–0.67)	**< 0.001**	0.48 (0.23–1.01)
Sex orientation			
Homosexual	0.73 (0.55–0.98)	**0.033**	0.60 (0.39–0.93)
Heterosexual	2.05 (0.56–7.44)	0.275	4.11 (0.87–19.53)
Forced sex	0.94 (0.64–1.37)	0.753	0.65 (0.34–1.21)
Condom use (30 days)	1.32 (0.94–1.85)	0.110**	1.99 (1.21–3.30)

*Ref*: Depression: no depression, age: < 20, education: never, employment: yes, residence: alone, gender: transgender, sexual orientation: bisexual, forced sexual intercourse: no, condom use: no

**= significant after RDS adjustment

*= not significant after RDS adjustment

The bolded *p* values implies significance

## Discussion

This study estimated that nearly one in two MSM in Cote d’Ivoire experience depression, which is consistent with other studies conducted in countries across Sub Saharan Africa [5, 58]. The results further demonstrated that the odds of health care utilization were significantly increased among MSM who screened positive for depression. Importantly, the relationships between depression and health care use held after adjusting for health care-related stigma. Depression may therefore serve as an outcome of health care stigma resulting from health care usage [59], with those who have visited a health care facility to seek care more likely to experience health care stigma and are also more likely to screen positive for depression. Stigma is known to decrease the coverage of evidence-based services for MSM by both limiting the provision of these services and ultimately, the uptake of available services [33]. Moreover, stigma is a known cause of psychological stress suggesting the need for health promotion interventions to address stress management and mental health. Finally, the strongest predictor of having engaged in sexual health services was knowledge of available services, consistent with studies across Sub-Saharan Africa as well as China [60–62].

Similar to many Sub-Saharan countries, the services specifically developed for MSM remain largely externally financed including stand-alone services. The challenge with this model is that there is limited coverage of education about specific sexual health services for MSM if one is not connected to these communities highlighting the importance of leveraging new technologies in promoting comprehensive sexual education. In Tanzania, a study by Dangat et al. found that the most common source of information on sexual education reported was through radio [63]. Overall, given the limited likelihood of the use of sexual health services observed here, comprehensive dissemination strategies are needed to drive awareness, interest, and uptake among those in need [64]. This was also reinforced with the results here that students and unemployed MSM were less likely to seek sexual healthcare services. Students, particularly college level, may have some knowledge about STI/HIV but limited self-efficacy about using condoms during anal sex given the limited specific education. [65, 66]. Sex work remained common in this study sample. And while the relationships between sex work and HIV outcomes can be complex given resiliency and community support from within the sex work community, there is potential for increased HIV acquisition risks through increased sexual partnerships and challenges in condom negotiation [63, 67–69]. These limited economic opportunities and ultimately unstable housing secondary to social stigmas reported here has also previously been shown to be related to engagement in care [70].

Depression and substance use including alcohol use are also common comorbidities of mental health among all adults [71]. Furthermore, alcohol use has also been shown to increase likelihood of STIs through increased reporting of condomless sex or sex with partners of unknown status [18, 72]. However, there was no association here between alcohol intake and utilization of sexual health care services in the past 12 months in either the unadjusted or adjusted models. One challenge here may have been the sensitive assessment of alcohol use, but it may have also been that people reporting misuse of alcohol were less likely to attend health clinics even if referred, consistent with earlier studies [73, 74]. Those who identified as gay/homosexual versus bisexual were significantly less likely to utilize healthcare services. The homonegative perception of same-sex practices and relationships both in Sub-Saharan Africa and in many countries of the world often manifests in rejection and discrimination affecting MSM. The goal is for these services to be implemented with fidelity and to be rights affirming, but the high levels of health-care related enacted stigma suggests that this is often not the case resulting in less engagement among those MSM who may be more open about their sexuality. [51, 75]. As noted, resiliency is part of the path forward, but as is continuing to improve the service delivery through early interventions for providers ideally as part of their core training curricula. [51, 52]. In addition to sexual practices, gender identity was significantly associated with engagement in services with cisgender men reporting lower levels of engagement in sexual health services. Transgender women tend to be at the highest risk of stigma given multiple intersecting stigmas related to gender identity, sexual practices, and potentially HIV resulting in limited opportunities [76, 77]. In the least, these data suggest the need for increased competence towards diverse gender identities and gender-related stigmas for providers to support effective engagement in care [78]. Ideally, people should be encouraged to seek appropriate sexual health services and to place value in appropriate management of HIV and STI as an important part of their healthcare services [79]. However, this assumes that these services will be both evidence-based and human rights affirming. Given that this is often not the case, resiliency to stigma may support seeking care including mediated by strong social support [80] or social capital [80]. The data here highlighted that those who had tested positive for both STIs and HIV were more likely to seek services which underscores the importance of leveraging this engagement to provide comprehensive services.

This study has a number of limitations. Due to the cross-sectional nature of the data collected, temporality cannot be assessed, and therefore a causal relationship between depression and sexual healthcare service utilization cannot be established. However, depression was measured over two weeks and utilization of sexual healthcare services in a one-year period, indicating it may be likely that healthcare utilization preceded depression in these analyses. The intersectionality of mental health in general, stigma and HIV-related stigma is complex and cross-sectional data may be limited in establishing these relationships. Future studies should consider establishing this temporality through prospective data collection. Also, topics on sexuality, STIs and stigma are culturally sensitive and hence may be underreported secondary to social desirability bias. Depression has also been found to be underreported in many African settings because of social stigmas related to depression and mental health more broadly [10].This social desirability bias may have also led to underreporting of hospital visits for HIV or STI services [4].

## Conclusion

Depression is prevalent among MSM in Cote d’Ivoire and associated with greater health care utilization presenting an opportunity to address the mental health needs of MSM. However, MSM with increased healthcare utilization also appear more likely to report experiences of stigma in health care settings secondary to their sexual orientation. Taken together, these data suggest the need to address stigma reduction or mitigation interventions, and better integrated mental and sexual health services are needed and may ultimately be key to reducing health disparities among MSM in Cote d’Ivoire. Integrating education about the specific needs of MSM into health care training curricula may also represent a strategy to normalize diverse sexualities and genders in Cote d’Ivoire. Ultimately, the sexual health and mental health needs are clear and so too is the intense and intersecting stigmas affecting engagement in services for MSM in Cote D’Ivoire. And the way forward includes implementing at scale evidence-based and human rights affirming strategies that will improve these services and ultimately decrease health disparities.
